# Reef-building corals thrive within hot-acidified and deoxygenated waters

**DOI:** 10.1038/s41598-017-02383-y

**Published:** 2017-05-26

**Authors:** Emma F. Camp, Matthew R. Nitschke, Riccardo Rodolfo-Metalpa, Fanny Houlbreque, Stephanie G. Gardner, David J. Smith, Marco Zampighi, David J. Suggett

**Affiliations:** 1 Climate Change Cluster, University of Technology Sydney, PO Box 123, Broadway, NSW 2007 Australia; 2 Institut de Recherche pour le Développement, Centre IRD de Nouméa, ENTROPIE (UMR250), BP A5, 98848 Nouméa cedex, New Caledonia; 3 School of Biological Sciences, University of Essex, Wivenhoe Park, Colchester, Essex CO4 3SQ UK

## Abstract

Coral reefs are deteriorating under climate change as oceans continue to warm and acidify and thermal anomalies grow in frequency and intensity. *In vitro* experiments are widely used to forecast reef-building coral health into the future, but often fail to account for the complex ecological and biogeochemical interactions that govern reefs. Consequently, observations from coral communities under naturally occurring extremes have become central for improved predictions of future reef form and function. Here, we present a semi-enclosed lagoon system in New Caledonia characterised by diel fluctuations of hot-deoxygenated water coupled with tidally driven persistently low pH, relative to neighbouring reefs. Coral communities within the lagoon system exhibited high richness (number of species = 20) and cover (24–35% across lagoon sites). Calcification rates for key species (*Acropora formosa*, *Acropora pulchra*, *Coelastrea aspera* and *Porites lutea*) for populations from the lagoon were equivalent to, or reduced by *ca*. 30–40% compared to those from the reef. Enhanced coral respiration, alongside high particulate organic content of the lagoon sediment, suggests acclimatisation to this trio of temperature, oxygen and pH changes through heterotrophic plasticity. This semi-enclosed lagoon therefore provides a novel system to understand coral acclimatisation to complex climatic scenarios and may serve as a reservoir of coral populations already resistant to extreme environmental conditions.

## Introduction

Atmospheric CO_2_ levels have nearly doubled since the industrial revolution resulting in unprecedented climatic change. Increased atmospheric heat trapping and CO_2_ absorption by seawater has driven ocean warming and acidification, which are already severely impacting coral reef ecosystems^1^. Warming elevates corals closer to their thermal thresholds^1^ and intensifies El Niño anomalies^2^ that drive mass bleaching events. In parallel, increased seawater acidity generally drives reduced coral calcification^3^ and/or accelerated reef and sediment dissolution^4, 5^. Many experiments and observations have examined the combined impact of warming and ocean acidification, but still largely fail to account for natural oscillations of these factors inherent to coastal systems, which are predicted to amplify under future climates^6^. Furthermore, such systems are expected to become increasingly deoxygenated as ocean warming and acidification alter thermal stratification, gas solubility, and biological metabolic activity^7^. How corals are influenced by this “deadly trio” of warming, acidity and deoxygenation within the next 50–100 years remains unexplored.

Corals populating the periphery of their optimal niche are likely acclimatised and/or adapted to relatively extreme growth conditions^8^, and have therefore become popular models to predict the structure and functioning of future reefs. Natural systems enable the study of coral communities free from the constraints of *in vitro* experimentation; notably the timescales (months to centuries) required for steady-state community-scale reorganisation to the prevailing extremes. No single system is a ‘perfect’ analogy for conditions predicted under future climates^9–11^ and ecosystem responses are not consistent across all extreme natural systems, most likely reflecting the different combinations of stressors at play. For example, hot and/or acidified semi-enclosed reefs can sustain high coral richness and cover to provide compelling evidence that coral populations can be resistant to one or more stressors^8, 12–14^. Semi-closed lagoons of Palau particularly harbour a rich and diverse coral community at seawater pH close to values projected for the end of this century^10, 12, 14^. Furthermore, corals thriving in rock pools of American Samoa have been shown to have both acclimatised and genetically adapted to warmer seawater^8^. Thus, understanding ecosystem scale re-organisation under climate change will inevitably only be achievable through a more collective assessment of naturally extreme coral systems. It is therefore imperative to identify additional and potentially unique study sites where multiple stressors operate, and within the context of natural variability of the main environmental parameters predicted for future reefs. Here, we describe a semi-enclosed lagoon system surrounded by mangroves (Bouraké, New Caledonia), where diverse and relatively abundant coral populations persist under the combined stress of elevated temperature, low pH and low dissolved oxygen, periodically fluctuating according to tidal and diurnal cycles. As such, it arguably represents the most suitable natural analogue to future extreme conditions documented to-date, and a unique platform to examine phenotypic acclimatisation and adaptive shifts to hot, acidified, and deoxygenated seawater.

**Figure 1:**
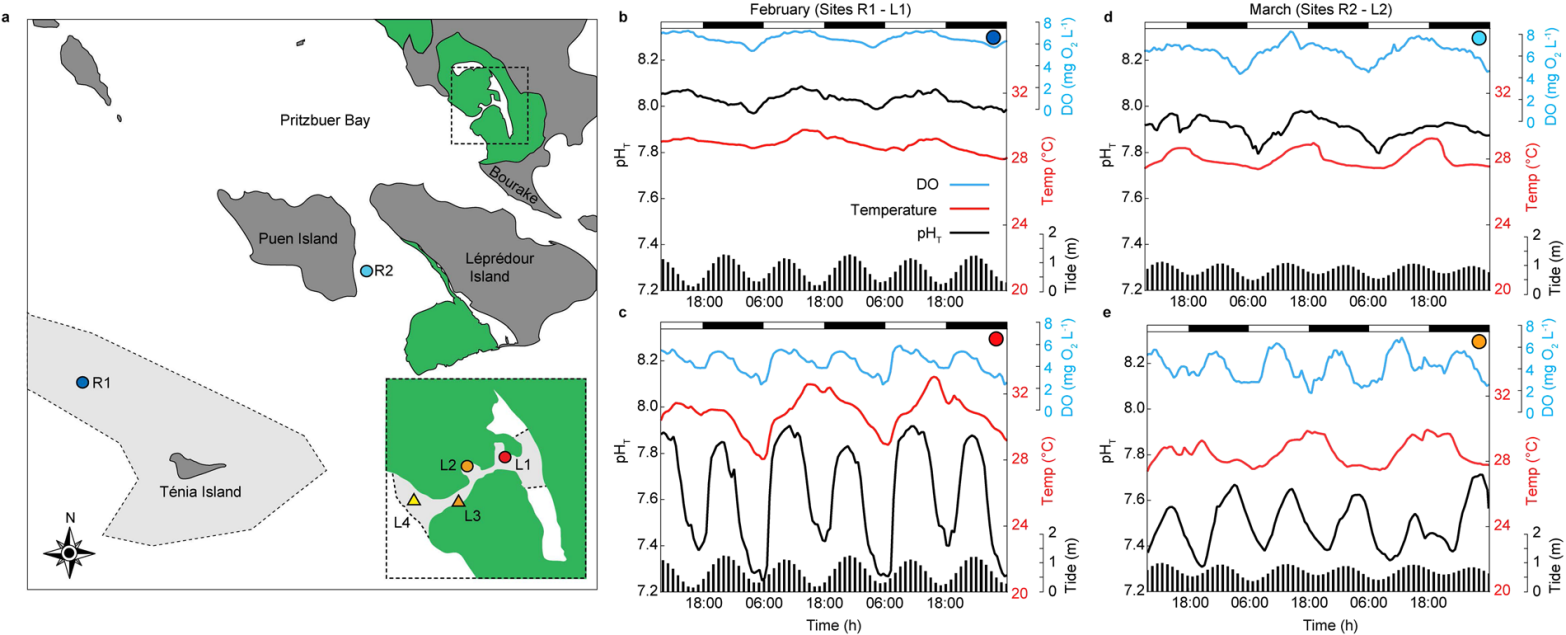
Map of the semi-enclosed lagoon system in Bouraké (New Caledonia) and reference reef sites along with their physico-chemistry. (**a**) Location of the study sites L1–L4 (shown in insert) inside the mangrove system (green areas), and reference reef sites R1, which is characterised as an exposed outer-reef (indicated by grey hashed areas), and R2, sheltered between two islands (indicated by dark grey areas). (**b**–**e**) Physico-chemical parameters measured in February for sites R1 (**b**) and L1 (**c**), and March for sites R2 (**d**) and L2 (**e**). pH in total scale (pH_T_), dissolved oxygen (DO, mg L^−1^), and temperature (°C) were coupled to the tidal cycles (x axes, bottom; vertical bars) and daily light cycles (x axes, top; horizontal bars). The base data for the map (**a**) were collected from map tiles at www.openstreetmap.org (© OpenStreetMap contributors, www.openstreetmap.org/copyright) under the Creative Commons Attribution-ShareAlike 2.0 licence (http://creativecommons.org/licenses/by-sa/2.0/), and customized in Adobe Illustrator (version 16).

The lagoon was subjected to no freshwater catchment input and a semi-diurnal tidal cycle (1.2 ± 0.3 m). The lagoon mouth led to a channel (*ca*. 4–5 m wide, 2–6 m depth) bordered by a shallow coral reef platform running through the lagoon to connect a series of sheltered shallow (1–2 m depth) bays. Four sites (L1–L4) were selected inside the lagoon, from the innermost bay to the lagoon mouth (Fig. 1, Supplementary Video 1). Two adjacent shallow (1–3 m depth) reef reference sites were chosen outside the lagoon: R1, representative of an exposed outer-reef subject to continuous open-ocean water, and R2, characteristic of an inner-reef sheltered between two islands with restricted water movement. We characterised the benthic composition (i.e. coral and abiotic substrate), seawater carbonate chemistry, and investigated the metabolic responses of dominant coral species, within and outside the lagoon, over three sampling periods (February-June 2016, see Methods). Our first sampling period anticipated a mass bleaching event, which affected almost all fringing reefs around the New Caledonian coast. Some corals were displaying early signs of bleaching both inside and outside of the lagoon; notably however, corals inside the lagoon were visibly recovered by our second sampling period in March 2016 (Supplementary Video 2, R.R.-M. pers. observations), while outside the lagoon the phenomenon progressed toward a significant percentage (80–90%) of corals bleached.

The lagoon waters were significantly warmer, acidified, and deoxygenated (Supplementary Table 1) relative to both reference sites across sampling periods (Fig. 1, Supplementary Figure 1, Table 1). For instance, during the warmest period in February, corals at L1 were exposed to highly variable and elevated temperatures (>33.0 °C), reduced pH (<7.3 pH_T_), and low dissolved oxygen levels (<2.3 mg L^−1^) compared to the reference sites. Diel variance of temperature, pH and oxygen was greatest within the lagoon, and temperature profiles corresponded to solar heating (*p* < 0.001, *R* ^*2*^ = 0.64 and 0.78 for L1 and L2 respectively), whereas daily pH_T_ (*p* < 0.001, *R* ^*2*^ = 0.65 and 0.30 for L1 and L2 respectively) and oxygen profiles (*p* < 0.001, *R* ^*2*^ = 0.37 and 0.18 for L1 and L2 respectively), corresponded with tidal flushing (Supplementary Figs 1, 2 and 3 and Supplementary Table 2).

**Table 1:** Data from the semi-closed lagoon of Bouraké (L1, L2), and adjacent reference reef sites (R1, R2).

Physico-chemical variables	Values	February 2016		March 2016	
		R1	L1	R2	L2
pH_T_	mean (±s.e.)	8.03 (0.01)	7.62 (0.02)	7.94 (0.01)	7.55 (0.01)
	max.	8.08	7.91	8.05	7.87
	min.	7.97	7.24	7.79	7.31
Temperature (°C)	mean (±s.e.)	29.0 (0.03)	30.9 (0.90)	28.1 (0.03)	28.4 (0.06)
	max.	29.9	33.1	30.2	30.8
	min.	28.1	28.1	26.6	25.9
Oxygen (mg L^−1^)	mean (±s.e.)	6.48 (0.04)	4.38 (0.08)	6.38 (0.04)	4.47 (0.06)
	max.	7.17	5.92	8.68	6.97
	min.	5.34	2.28	4.33	1.80
Salinity	mean (±s.e.)	35.4 (0.02)	36.2 (0.07)	34.2 (0.03)	34.4 (0.02)
*A*_T_ (µmol kg^−1^)	mean (±s.e.)	2306 (7.13)	2268 (5.48)	2246 (5.84)	2272 (7.41)
Ω_arg_	mean (±s.e.)	3.76 (0.02)	2.02 (0.07)	2.99 (0.02)	1.47 (0.02)
*p*CO_2_ (µatm)	mean (±s.e.)	413 (2.91)	1399 (67.89)	527 (3.51)	1531 (23.59)
HCO_3_^−^ (µmol kg^−1^)	mean (±s.e.)	1730 (2.85)	1398 (10.58)	1788 (2.26)	2048 (3.10)
CO_3_^2−^ (µmol kg^−1^)	mean (±s.e.)	233 (1.14)	124 (4.27)	185 (0.90)	91 (1.25)

Data was collected over three days in February 2016 at the lagoon site L1 and reference site R1, and during eight days in March 2016 at L2 and R2. Total alkalinity (*A*_T_) was determined from discrete water samples (see Methods, *n* = 9 and 6 for R1, R2, *n* = 6 and 3 for L1, L2). For the remaining variables, *n* = 138 for R1 and L1; *n* = 408 for R2 and L2).

Across the possible diel and tidal cycle combinations for the Bouraké lagoon (see Methods), salinity normalised dissolved inorganic carbon (*nC*_*T*_) to total alkalinity (*nA*_*T*_) plots were generated to assess the dominant mechanisms influencing the carbonate chemistry^15^. A system where calcification and dissolution are dominant processes has a linear regression slope approaching 2. For the Bouraké lagoon the *nA*_*T*_ *-nC*_*T*_ slopes were 0.60 and 0.62 for sites L2 and L4 respectively, and both lagoon sites showed strong correlation between *nA*_*T*_ and *nC*_*T*_, with *R* ^2^ > 0.7 (Supplementary Figure 4). Using the slopes of the *nA*_*T*_ *-nC*_*T*_ plots, net ecosystem calcification to net community production (NEC: NEP) was calculated to be 0.44 (±0.007). Thus whilst the Bouraké lagoon is influenced by photosynthesis-respiration (and thus CO_2_ uptake/release), co-variability between n*A*_*T*_ and n*C*_*T*_ strongly also indicates significant influence from calcification and dissolution and possible nitrate and sulphate reduction/oxidization^15^. Overall variance in *A*_T_ throughout the day was large (Supplementary Table 3), and comparable to other shallow reef systems^16^.

Influx of seawater with pH_T_ of around eight from outside the Bouraké lagoon on the flooding tide resulted in a large pH_T_ increase of 0.442 units over an hour at the mouth of the lagoon (site L4, Supplementary Figures 5 and 6). Seawater in the lagoon over slack tide experienced reduced pH_T_, with lowest pH_T_ recorded at low tide that corresponded also with increased *A*_*T*_ (Supplementary Figures 5 and 6). At low tide, respiration/CO_2_ invasion, carbonate dissolution and potential contribution from nitrate and sulphate reduction were dominant processes, with data points on the *nA*_*T*_ *-nC*_*T*_ plots clustered in the top right quadrants (*see* Supplementary Figure 4). Together the *A*_T_ and pH_T_ variance within the lagoon resulted in *p*CO_2_ and aragonite saturation (Ω_arg_) that also varied with tidal state (Supplementary Figures 5 and 6). Salinity was comparable to the reference sites (Table 1), and the mean irradiance was 23.6% lower via tidal sediment re-suspension, but still reached intensities observed for the reference sites (~2,000 µmol photons m^−2^ s^−1^ at midday).

The Bouraké lagoon is characterised by dynamic physico-chemistry, however, diel and tidal control of the system provides a regular and predictable environmental history (Fig. 1). Consequently, this system overcomes the element of uncertainty that accompanies more stochastic extreme sites (e.g., variable CO_2_-dosing at vents refs 9, 11, 13, 17 and 18) used as analogous conditions to predict coral reef community shifts under predicted ocean acidification levels. For example, in Mexico, natural groundwater discharge of acid freshwater (i.e. pH down to 6.7 units) limits the occurrence of scleractinian corals in the vicinity of the submarine springs^19^. However, natural acidification at this site is spatially limited and temporally fluctuating, therefore limiting conclusions at the reef community scale. Volcanic CO_2_ vents have been used as the best natural analogues^11^ since the effect of submarine CO_2_ emissions alter the seawater carbonate chemistry of the surrounding seawater in a relatively large area (i.e. tens to hundreds of meters). Vent sites have revealed reduced coral diversity^9^ as well as shifts in the community composition from hard to soft corals^17^ or macroalgae dominance^18^ due to ocean acidification; it is noteworthy that at these natural sites the seawater carbonate chemistry is highly variable, and more importantly not predictable.

In the Bouraké lagoon, seawater pH, temperature and oxygen change according to predictable diel-tidal cycles (Fig. 1). As a result, corals spent 44% of time at pH_T_ of 7.7–7.8 and 71% of time at temperatures predicted for the end of the century under Intergovernmental Panel on Climate Change (IPCC) scenario RCP4.5 (relative to the mean temperature of site R1, Supplementary Figure 7). Such environmental predictability promotes phenotypic plasticity, and the associated fitness benefits facilitate acclimatisation^20^. Other natural low-pH systems such as CO_2_ vents generally exhibit low coral cover^9, 17, 18^ and loss of complexity^9^ of scleractinian corals, with the exception of the Palauan reef where relatively abundant and diverse coral communities have been described^10, 12, 14^. Seawater carbonate chemistry was measured inside and outside the Palauan system, and from the shore to the barrier reefs. Measurements showed a gradient of Ω_arg_ from 3.2 to 3.8 close to the reef, which is within the normal variability of coral reef lagoons^21^. Lower levels of acidification (Ω_arg_ from 2.4 to 3.1) were only found at the large, 1.5 × 1.3 miles, semi-closed Nikko Bay^14^ where restricted circulation and biological activity contribute to elevate the seawater *p*CO_2_, which is similar to the Bouraké lagoon.

**Figure 2:**
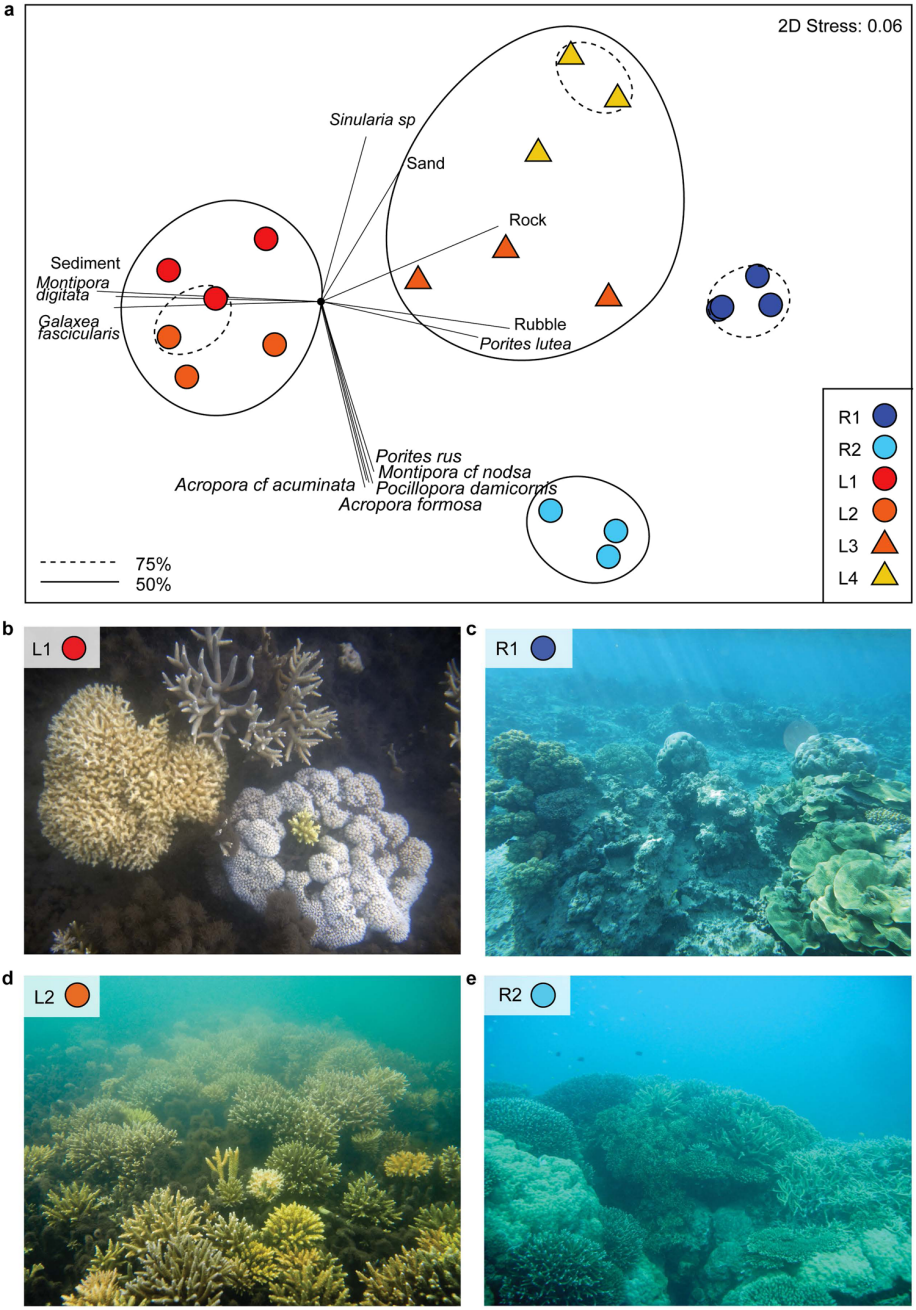
Benthic composition of the Bouraké semi-enclosed lagoon system and reference reef sites. (**a**) A multidimensional-scaling plot of benthic data per sites with 50% (solid) and 75% (hashed) similarity shown. Pearson’s correlations exceeding *R* > 0.6 between benthic taxa are represented as vectors in black. (**b–e**) Typical benthic scenes of the lagoon sites L1 (**b**) and L2 (**d**), the exposed reference reef site R1 (**c**), and sheltered reference site R2 (**e**).

Despite the extreme physico-chemical conditions of the Bouraké lagoon, scleractinian relative coral abundance was generally high (26.7–34.6%, sites L1–L3) and comparable to the reference sites (10.3–55.0%; Supplementary Table 4). Coral diversity was also similar to the reef at sites L2 and L3 (Shannon’s H Index, Reef 1.22 ± 0.4, Lagoon 1.27 ± 0.1), while reduced at sites L1 and L4 (Shannon’s H Index, 0.58 ± 0.1). The benthic framework of the Bouraké lagoon was highly heterogeneous (Fig. 2, Supplementary Figure 8), where the sheltered bays (L1 and L2) were predominantly comprised of sediment (66.3 ± 2.5%), whereas the lagoon mouth (L4) and channel (L3) were dominated by rock (20.1 ± 1.7%) and rubble (9.8 ± 2.5%; Supplementary Table 4). Unconsolidated sediments were stabilised by the mangrove roots, and coral colonies were generally established on dead coral framework (Supplementary Figure 9), rather than directly on the mangrove roots as described in other systems^22^.

A total of 20 common scleractinian coral species were identified within the Bouraké lagoon, ranging in growth form and family, with all but two species found on the reference sites (Supplementary Table 5). The sheltered bays (sites L1 and L2) and coral platforms (site L3) of the Bouraké lagoon were dominated by architecturally important reef-forming species of Acroporidae, Poritidae, and Montiporidae (Fig. 2, Supplementary Video 1). Only at the exposed lagoon mouth (L4) was scleractinian cover low (5.8 ± 0.8%), comprised of mono-specific representatives of the genera *Favites* and *Porites*, and instead dominated by soft corals (46.7 ± 11.1% cover; Supplementary Table 4). As variance in pH_T_, temperature and oxygen was similar across lagoon sites (Table 1), the reduced scleractinian coral cover and absence of branching species at L4 clearly reflects the interaction of other factors. For example, maximum current speed associated to the tidal fluxes through L4 was *c*.*a*. 0.5 ms^−1^. As low pH conditions compromise physical integrity (particularly branching taxa), including coral skeletal density^10, 12^ and cementation^23^, the dynamic nature of L4 may exceed establishment thresholds of less-robust scleractinian coral species. Similarly, supra-optimal flow can reduce feeding capacity of taxa reliant on heterotrophy^24^, however the optimal conditions for food capture of corals under the Bouraké lagoon conditions must be determined in future studies.

**Figure 3:**
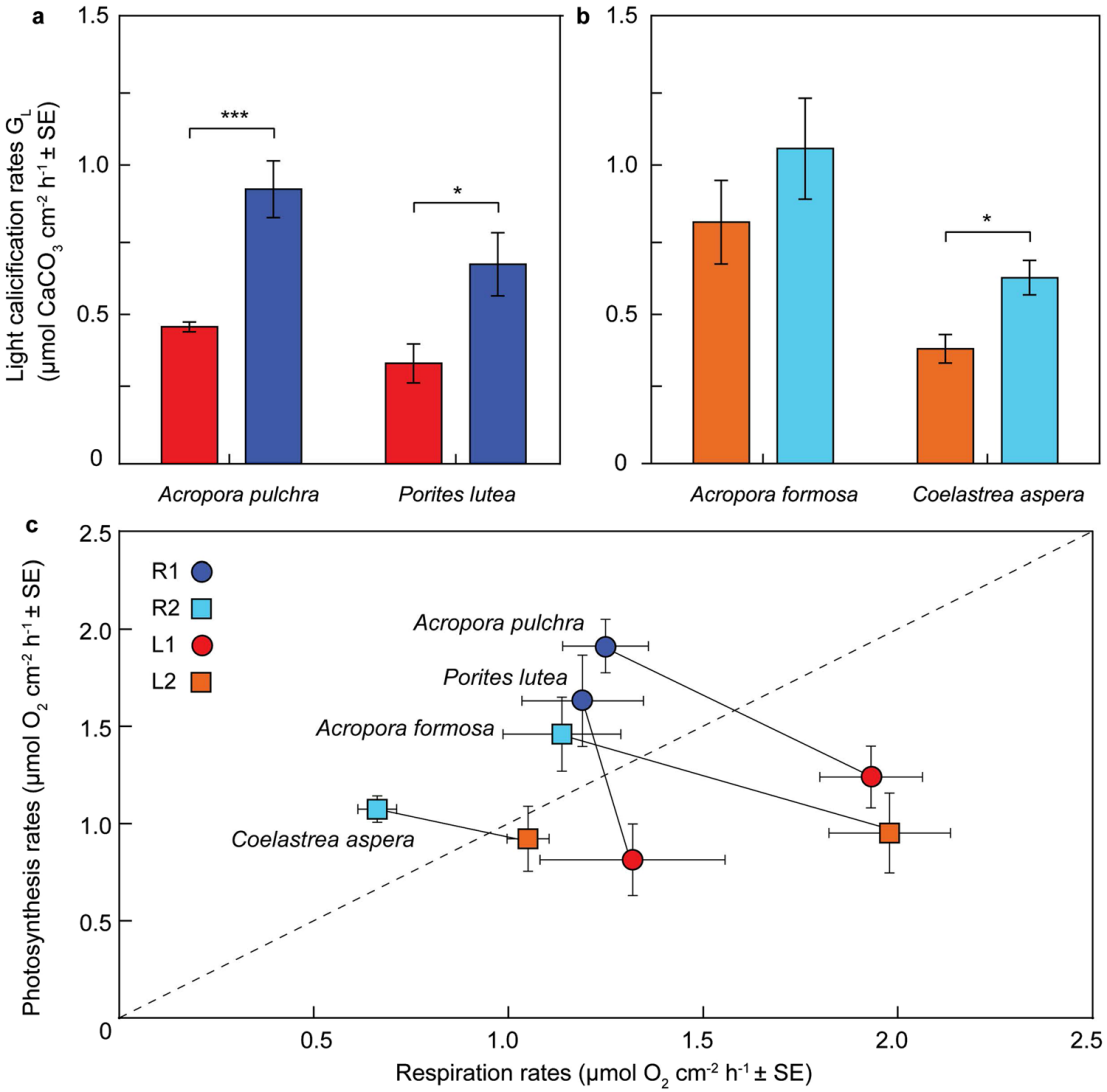
Coral metabolism in the Bouraké lagoon system *versus* reference reef sites. (**a**,**b**) Light-driven calcification (G_L_) for *Acropora pulchra* and *Porites lutea* (**a**) at lagoon site L1 (red bars) and reference site R1 (﻿dark blue bars), and *Acropora formosa* and *Coelastrea aspera* (**b**) at lagoon site L2 (orange bars) and reference site R2 (light blue bars). *p < 0.05, **p < 0.01, ***p < 0.001 between groups according to t-test analysis (*n* = 4 per species). (**c**) The metabolic comparisons of photosynthesis and respiration rates between reference sites R1, R2 versus lagoon sites L1, L2. Species-specific metabolic shifts are indicated by lines (black) and the dashed lines represent the 1:1 ratio.

Metabolic activity of corals across sites (*Acropora pulchra* and *Porites lutea*, sites R1 and L1; *A*. *formosa* and *Coelastrea aspera*, sites R2 and L2) generally demonstrated significantly higher light-driven calcification (G_L_) rates for the reference sites relative to lagoon populations (mean G_L_, 0.82 and 0.50 µmol CaCO_3_ cm^−2^ h^−1^ respectively; *p* < 0.05 except for *A*. *formosa*; Fig. 3, Supplementary Tables 6 and 7). Dark calcification was also reduced for the lagoon corals, but to a lesser extent than light-calcification (Supplementary Tables 6 and 7). Reduced calcification (*ca*. 30–40%) of the lagoon corals relative to the reference sites is consistent with *ca*. 38% lower rates of photosynthesis (P) in part reflecting the reduced light availability. Furthermore, reductions in Ω_arg_ of the lagoon system relative to the reef could also explain reductions in calcification. In natural systems^25^ and laboratory studies e.g.^26, 27^ where temperature and pH have been considered representative of future climate-scenarios, coral calcification has decreased by a similar proportion, corresponding to a loss of photosynthetic activity but unchanged respiration (R) (thus lower P:R). We similarly observed lower P:R for coral populations in the Bouraké lagoon (0.6–0.8) compared to the reference sites (1.3–1.6; Fig. 3); however, this was driven predominantly by an increase in respiration (11–74%) and to a lesser extent a reduction in photosynthesis (14–50%).

Up-regulation of respiration by the lagoon coral populations was consistent with a higher loading of organic carbon content in the sediment (15.5 ± 2.1 mg g^−1^) and hence greater heterotrophic potential compared to the reefs (4.41 ± 0.2 mg g^−1^; Supplementary Table 8).

A shift from autotrophy to heterotrophy has been evidenced under high-sediment/reduced light-conditions^28^ and elevated temperatures^29^, both characteristic of the Bouraké lagoon. The coral *Porites lutea* has also demonstrated the ability to acclimate to high environmental variance (specifically temperature and pH) by the modulation of mixotrophic nutrition^30^. Furthermore, the adverse effects of high *p*CO_2_ on coral calcification have reported to be off-set by enhanced feeding for some^31, 32^, but not all^33, 34^ coral species. Our observations of higher particulate sediment and greater carbon content in the lagoons (Supplementary Table 8) support the hypothesis that food supply confers corals with resistance to elevated acidification^35^. Corals employ a variety of mechanisms, (e.g. contact with discharged nematocysts, tentacular grabbing, and mucus adhesion) to obtain a wide range of food sources encompassing dissolved and particulate organic matter and live particulate organic matter^36^. Surface sediment can retain significant quantities of bacteria, microbial exudates, protozoa, interstitial invertebrates, microbial algae and detrital organic matter, all of which are considered additional food sources for corals^37^. Experiments have shown active ingestion of sediment followed by digestion^38^, including suspended particulate matter and benthic sediments layered onto corals’ surfaces^39^. Further research is needed to establish the extent of heterotrophy within the Bouraké lagoon; even so, metabolic plasticity appears critical for corals to acclimatise to these natural extremes of low pH, high temperature and deoxygenation.

Our observations of well-established, diverse, and architecturally complex coral taxa in the Bouraké lagoon provides compelling evidence that diverse coral populations can persist (albeit with reduced calcification) under high-temperature (ranging from 25.9–33.1 °C), low-pH (ranging from 7.24–7.91) and deoxygenated waters (ranging from 1.80–6.97 mg L^−1^), relative to neighbouring reefs. Metabolic plasticity, through up-regulation of metabolic rates, appears to be a key mechanism for these coral populations to thrive under extreme environmental conditions. The Bouraké lagoon therefore provides a new model system to study and resolve the fitness trade-offs associated with acclimatisation to complex stressor interactions that are potentially indicative of future climates for reefs worldwide. Evidence here, and from other similar habitats increasingly highlight that reef neighbouring systems could act as local reservoirs of coral populations highly resistant to extreme environmental conditions.

## Methods

### Study sites and sampling strategy

The study was conducted at a semi-enclosed lagoon within a mangrove system (L1–L4, 21° 56.915 S; 165° 59.577E, Fig. 1) and at two adjacent reference reef sites (R1, 21° 59.784 S; 165° 54.992E; R2, 21° 58.189S; 165° 58.184E) in Bouraké, New Caledonia. Three sampling periods were undertaken to characterise the lagoon in comparison to the adjacent reefs (February, March, and May 2016).

### Environmental data

To characterise the physico-chemical conditions of each site, a suite of measurements were taken. SeaFET^TM^ pH loggers (30-min logging interval) were simultaneously deployed across comparative sites, for each sampling period to measure pH_T_ (total scale). Discrete water samples (from a depth of *ca*. 0.5 m) were taken at the start and end of SeaFET^TM^ deployments to corroborate the pH_T_ measurements. Water samples were collected in borosilicate bottles and immediately measured on the boat for pH_T_ using a pH probe (913, Metrohm) calibrated with TRIS buffer.

Salinity, oxygen and temperature were measured (30-min logging interval) using a sonde (600 OMS-M, YSI). In February, light (Lux) was measured (one-min logging interval) *in situ* at R1 and L1 using HOBO^®^ Pendant light loggers (Microdaq). In March, light (PAR) was also measured within the lagoon using a 4π spherical underwater quantum sensor (LI–193SA). In addition, total organic and inorganic content of marine sediment was determined for sites L1, L2 and R2 using the modified Walkley-Black^40^ and loss-on-ignition^40^ methods. The top 2 cm of sediment at each site was collected as per ref. 41.

Total alkalinity (*A*_T_) was determined from discrete water samples collected as described for the discrete pH measurements. Samples were fixed with HgCl_2_ and sealed. Samples were transported back to the Institut de Recherche (IRD) laboratory (Nouméa, New Caledonia) where *A*_T_ was determined using an autotitrator (848 Titrino, Metrohm), verified with certified reference materials distributed by A. Dickson (Batch 142, Scripps Institute of Oceanography). In February and March opportunistic diurnal (morning and afternoon) *A*_T_ samples were collected (R1 & L1 *n* = 9, R2 & L2 *n* = 6). From these data, it was apparent that the Bouraké lagoon experienced a large diel change in *A*_*T*_; thus, in May 2016, a two-day, high-resolution (every-hour from sunrise to sunset) sampling effort was performed to determine the extent of *A*_T_ variance over the tidal/diel cycles (L2 *n* = 13, L4 *n* = 14). For that, *A*_T_ values were used with the corresponding *in situ* pH_T_, temperature, salinity and depth (m) of each sampling periods to determine the remaining carbonate system parameters, using CO2SYS^42^, the dissociation constants of refs 43 and 44 for KHSO_4_, and ref. 45 for boric acid.

### Biological data and *in vitro* incubations

To characterise the benthic habitat of all sites, continuous-line intercept video-transects were conducted. Within each site, 3 × 50 m transects were randomly located. A high-definition video-camera was used along transects at a fixed distance. Benthic composition was quantified using the categories defined by ref. 46, while corals were identified to species level where possible.

During the sampling in February, *in vitro* coral incubations were conducted to establish rates of photosynthesis, respiration and calcification for four coral fragments (<5 cm length) of *Acropora pulchra* (sites R1 and L1), *A*. *formosa* (sites R2 and L2), *Porites lutea* (sites R1 and L1) and *Coelastrea aspera* (sites R2 and L2). Removed fragments (branching species) and small colonies (massive/sub-massive species) were carefully collected and transported in individual zip-lock bags to the laboratory within 1-h of collection. Samples were allowed to recover for 4-h after collection in aquaria using a recirculating water system containing seawater from the sites of collection at controlled light (240 µmol m^−2^ s^−1^) and temperature (within 0.5 °C of *in situ* habitat temperature) conditions. Lighting was provided through two aquarium led light spots (LD2–60 cm, Hopar), while temperature was controlled by a bar-heater and re-circulating pump.

Hourly rates of photosynthesis, respiration and calcification were determined for all samples through 1-h light and 1-h dark incubations. Before the incubation, any abiotic substrate that was not live coral tissue was carefully covered using parafilm to prevent any non-target biological alteration to the incubated seawater (*see* Supplementary Table 9). Samples were incubated in 250 mL sealed glass incubation chambers filled with seawater collected from the sites of collection and continuously mixed using stirring bars. Before initial experimentation, one colony of *A*. *pulchra* and one colony of *P*. *lutea* were incubated under the experimental conditions to check that the volume of water-to time ratio was correct; i.e. that a metabolic drift could be detected without anoxic or hypoxic conditions occurring^47^. Three chambers for each incubation were left without corals and used to correct the physiological measurements for metabolic microbial activity of the water for incubation. Incubation temperature was based on the mean *in situ* conditions of each habitat (see Table 1). Chambers were semi-submerged in a water bath containing a heater and pump to maintain the temperature as stable as possible (±0.5 °C) over the duration of the incubation. Temperature was monitored at the start and at 10-min intervals until the end of the incubation using a multi-meter and temperature probe (SenTix, WTW). pH_T_ was also measured at the start and end of incubations, with an average change ± S.E. of 0.14 ± 0.01. Incubation light intensities were determined by measuring the light saturation coefficient (*E*_k_) of the corals using pulse amplitude-modulated (PAM) flurometer (Imaging PAM, Max/K. RGB, Walz GmbH, Effeltrich, Germany, ref. 48). *E*_k_ values were similar between corals from different sites and a mean light level of 240 ± 3.2 µmol m^−2^ s^−1^ was used. Two aquarium lights (LD2–60 cm, Hopar) were used to create a standard light level measured with a Li–Cor 4π spherical underwater quantum sensor (LI–193SA). Dark conditions were created by black-out material placed over the incubation chambers.

Net calcification (G) rates were determined by the alkalinity anomaly technique^49^ in both the light (G_L_) and dark (G_D_). Difference in *A*_T_ between the start and end of each incubation period were corrected for any changes in *A*_T_ of the three seawater controls. Incubations were *ca*. 1-h with a 1-h dark transition time occurred before the dark incubation to allow rates to stabilise to dark conditions^47^. Normalised rates of calcification (µmol CaCO_3_ cm^2^ h^−1^) were calculated by standardising for chamber seawater volume, incubation time and coral surface area as:

1 $$G(t)=[\frac{({\rm{\Delta }}{A}_{T}\cdot \rho \cdot 0.5)\cdot V}{{I}_{t}\cdot SA}]/1000$$

where *A*_T_ = total alkalinity (µmol kg^−1^), *V* = volume of seawater (L) within the incubation chamber, I_*t*_ is incubation time, SA is the coral surface area (cm^2^), ρ is the density of seawater and 0.5 accounts for the decrease of *A*_T_ by two equivalents for each mole of CaCO_3_ precipitated. Surface area was determined by the Advanced Geometric Technique^50^. Net photosynthesis (P_N_) and respiration (R) were determined by changes in oxygen for each incubation chamber during the light and dark incubations respectively, corrected for any changes in oxygen of the three seawater controls. Oxygen was quantified using a multi-meter and oxygen probe (FDO, WDW) (accuracy 0.01 mg L^−1^). Rates were normalised as described for calcification (to give µmol O_2_ cm^2^ h^−1^):

2 $${P}_{N}\,and\,R(t)=[\frac{({\rm{\Delta }}{O}_{2})\cdot V}{{I}_{t}\cdot SA}]/1000$$

Gross photosynthesis (P_G_) was calculated by the addition of P_N_ and R.

### Statistical analysis

Temperature, oxygen, salinity and pH_T_ were analysed using a two-tailed *t*-test with Welch correction applied to oxygen data as it did not demonstrate homoscedasticity. To investigate the effect of tidal-cycle and time-of-day on each physico-chemical parameter, linear (y = bo + b_1_ *x*) models were fitted. Goodness of fit was determined from statistical significance (*p* < 0.05) and *R* ^2^. Cumulative time of physico-chemical variables (pH_T_, temperature and dissolved oxygen (DO) for the lagoon site L1 relative to Intergovernmental Panel on Climate Change (IPCC) estimates century under scenarios RCP 4.5 and RCP 8.5 were determined. We note that the IPCC models are based on open-ocean global averages, and use these as the only accepted models to illustrate projected variance for 2100.

The Shannon Diversity Index (*H*) was determined on the benthic transect data. Mean benthic covers of the dominant taxa were compared between sites using Kruskal-Wallis and *post-hoc* Dunn’s multiple comparisons test. Similarities between site benthic structures were compared using a multidimensional-scaling plot (MDS), on square-root transformed data, generated by a SIMPROF test. Clusters (50% and 75% similarity) were generated from a Bray-Curtis similarity matrix of all benthic data. The MDS vectors were generated by Pearson’s Correlations exceeding *R* > 0.6, between plot ordinations and benthic categories. Coral metabolic parameters (net light (G_L_) and dark calcification (G_D_), photosynthesis and respiration) were compared for species between sites using a *t*-test. All assumptions of normality and homoscedasticity were met; G_L_ was log-transformed to meet these assumptions. To assess the dominant mechanisms influencing the carbonate chemistry salinity-normalised (S = 36) A_T_ (*nA*_*T*_) to dissolved inorganic carbon (*nC*_*T*_) plots were generated^51^. The ratio of net ecosystem calcification to net community production (NEC:NEP) were derived from these *nA*_*T*_ *-nC*_*T*_ plots as:1/[(2 /m)-1] (where m is the regression coefficient from the corresponding linear equation of *nA*_*T*_ vs. *nC*_*T*_. Statistical analyses were conducted using Graphpad Prism^52^, R studio^53^, and PRIMER (version 6).

## Electronic supplementary material

Underwater footage from the Bouraké semi-enclosed lagoon system at site 2 (L2).

Underwater footage from Sainte-Marie Bay and the Bouraké semi-enclosed lagoon system at site 1 (L1) in March 2016.

Supplementary Information
