# Supplementary material for: Reef-building corals thrive within hot-acidified and deoxygenated waters
Source: Sci Rep. 2017 May 26;7:2434. doi: 10.1038/s41598-017-02383-y (PMC5446402; doi:10.1038/s41598-017-02383-y)
Supplement: Supplementary file 3 — Supplementary Information [file 41598_2017_2383_MOESM3_ESM.pdf]

Supplementary Information

Reef-building corals thrive within hot-acidified and deoxygenated waters

Emma F. Camp[1‡], Matthew R. Nitschke[1‡], Riccardo Rodolfo-Metalpa[2]*, Fanny Houlbreque[2], Stephanie G. Gardner[1], David J. Smith[3], Marco Zampighi[2], David J. Suggett[1]

[‡] EFC and MRN joint lead authors

*Corresponding author: riccardo.rodolfo-metalpa@ird.fr

Supplementary Information: Figures

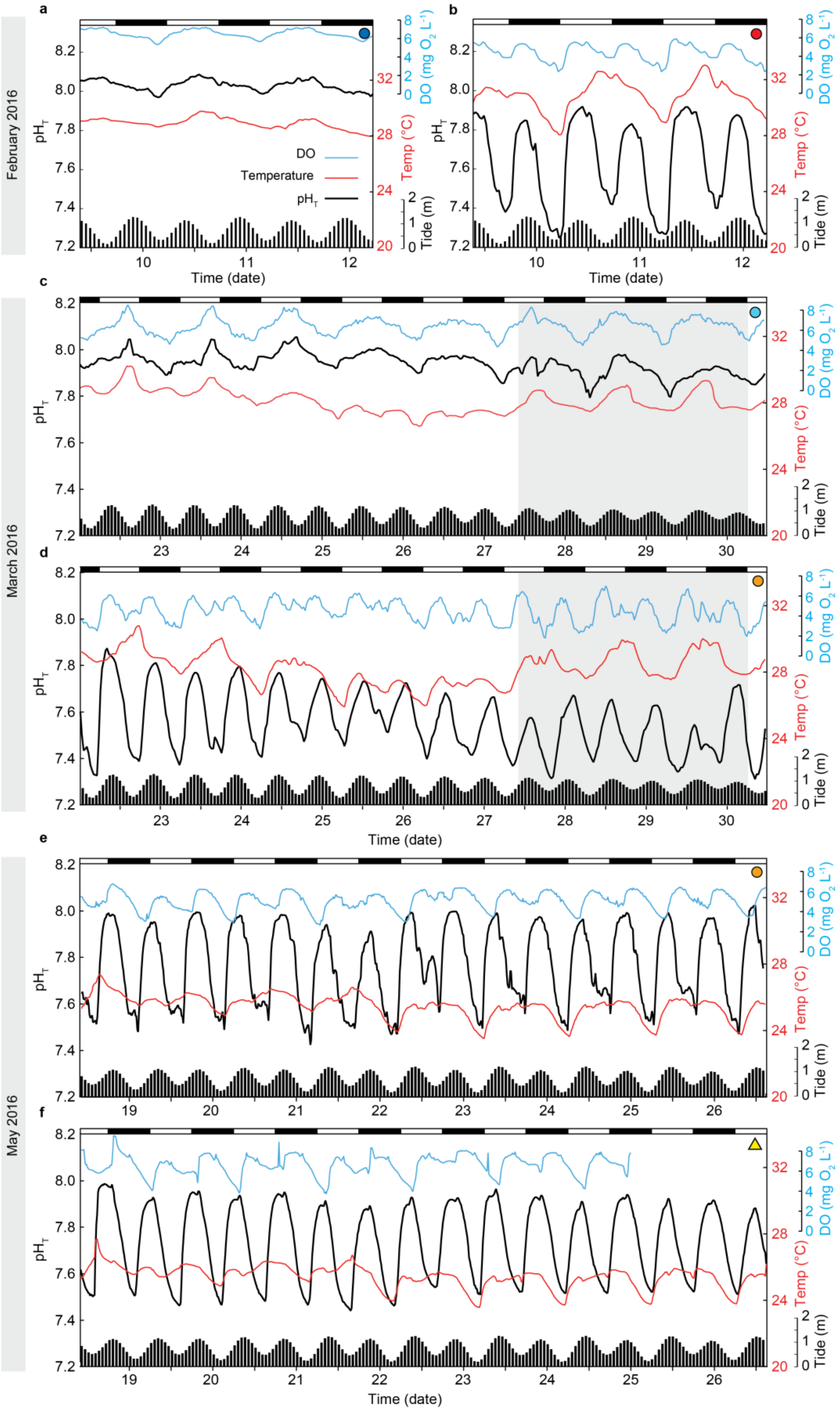

Figure S.1| Seawater physico-chemical data for all sites collected across February, March, and May (2016). Parameters measured include pH (total scale, pH_T), dissolved oxygen (DO, mg L^{-1}), and temperature (°C) over time (date). Sampling time points include: February for sites R1 (**a**) and L1 (**b**), March for sites R2 (**c**) and L2 (**d**), and May for sites L2 (**e**) and L4 (**f**). Data is coupled to the tidal cycles (x axes, bottom; vertical bars) and daily light cycles (x axes, top; horizontal bars).The shaded grey areas (**c** & **d**) indicate the data displayed in Figure 1.

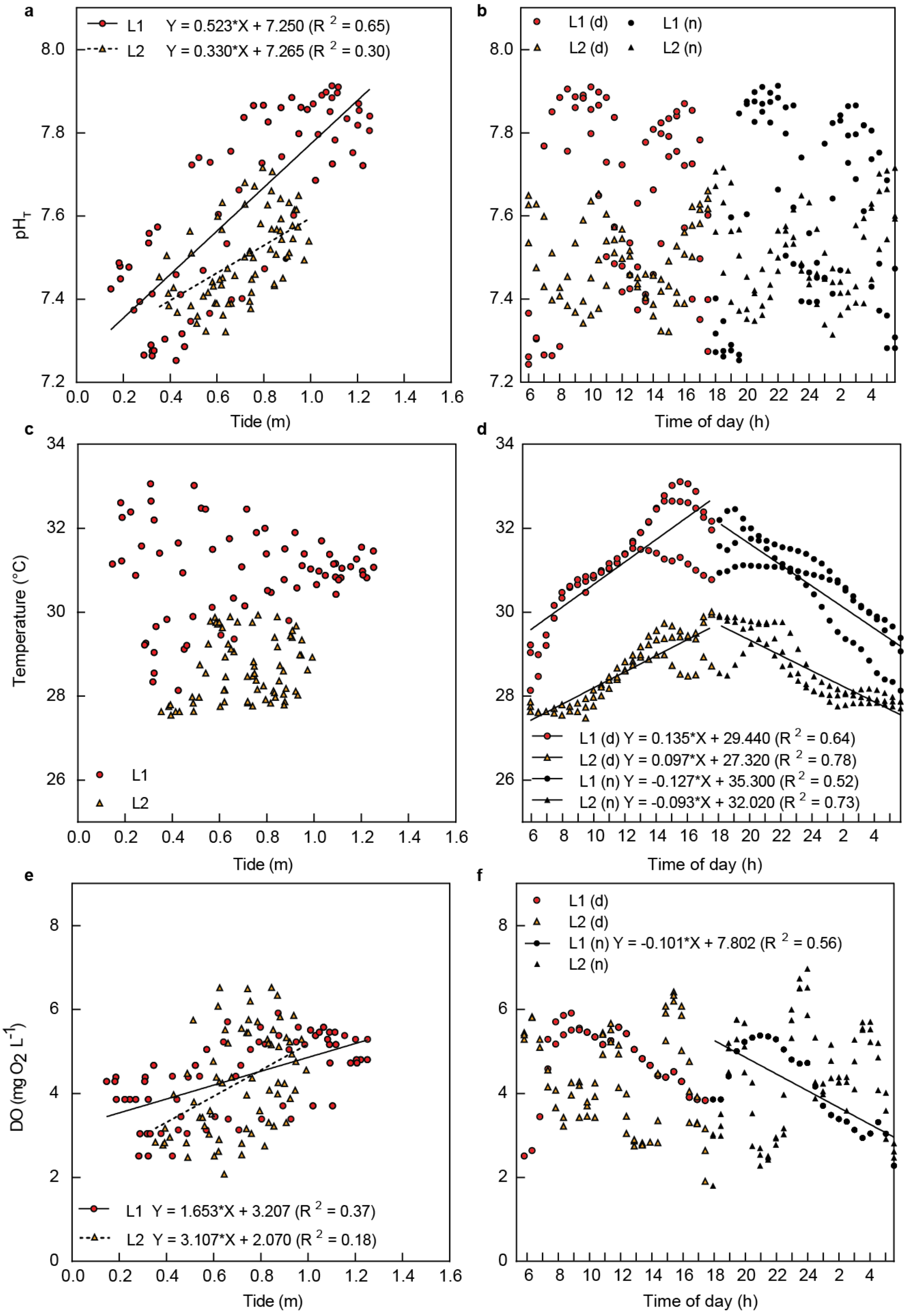

Figure S.2| Physico-chemical relationships with tide and time of day for the Bouraké lagoon sites. Parameters measured include total pH (pH_T; **a** & **b**), temperature (°C; **c** & **d**), and dissolved oxygen (DO, mg L^{-1}; **e** & **f**) against tide (m) and local time of day (h) for lagoon sites L1 (circles) and L2 (triangles). For time of day (**b**, **c**, and **f**), coloured symbols represent data recorded during the day and black symbols at night. Linear regressions significantly different from zero ($p < 0.05$) are indicated by lines with corresponding equations and R^2 values (see Supplementary Table 2 for full statistics).

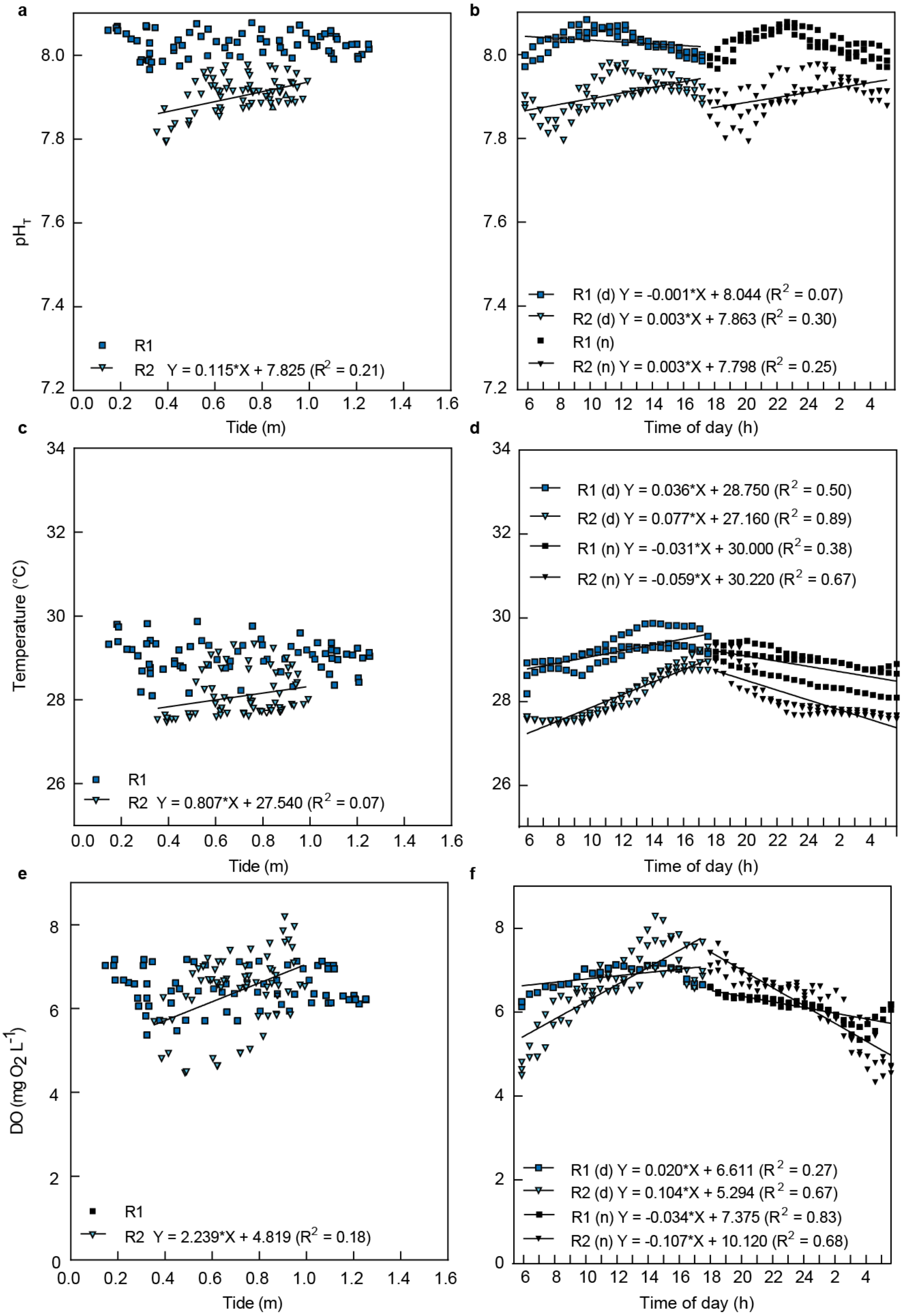

Figure S.3| Physico-chemical relationships with tide and time of day for reference reef sites. Parameters measured include total pH (pH$_T$; **a** & **b**), temperature (°C; **c** & **d**), and dissolved oxygen (DO, mg L^{-1}; **e** & **f**) against tide (m) and local time of day (h) for reference sites R1 (squares) and R2 (triangles). For time of day (**b**, **c**, and **f**), coloured symbols represent data recorded during the day and black symbols at night. Linear regressions significantly different from zero ($p < 0.05$) are indicated by lines with corresponding equations and R^2 values (see Supplementary Table 2 for full statistics).

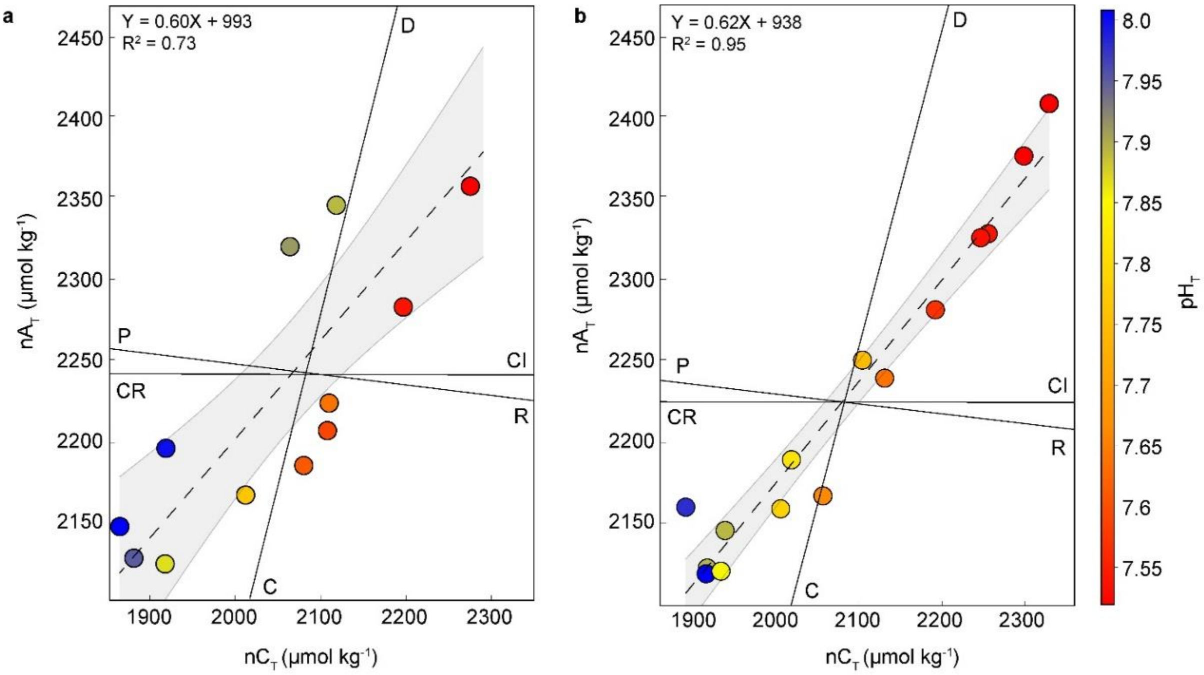

Figure S.4| Salinity-normalized (S= 36) total alkalinity (*nA_T*) and dissolved inorganic carbon (*nC_T*) plots with best-fit linear regression (hashed line) with 95% confidence bands for Lagoon sites L2 and L2 in May 2016. Black lines represent the theoretical impact of calcification (C), carbonate sediment dissolution (D), photosynthesis (P), CO_2 release (CR), CO_2 invasion (CI) and respiration (R) on nA_T and nC_T. C and D are dominant processes when a linear regression slope approaches 2.

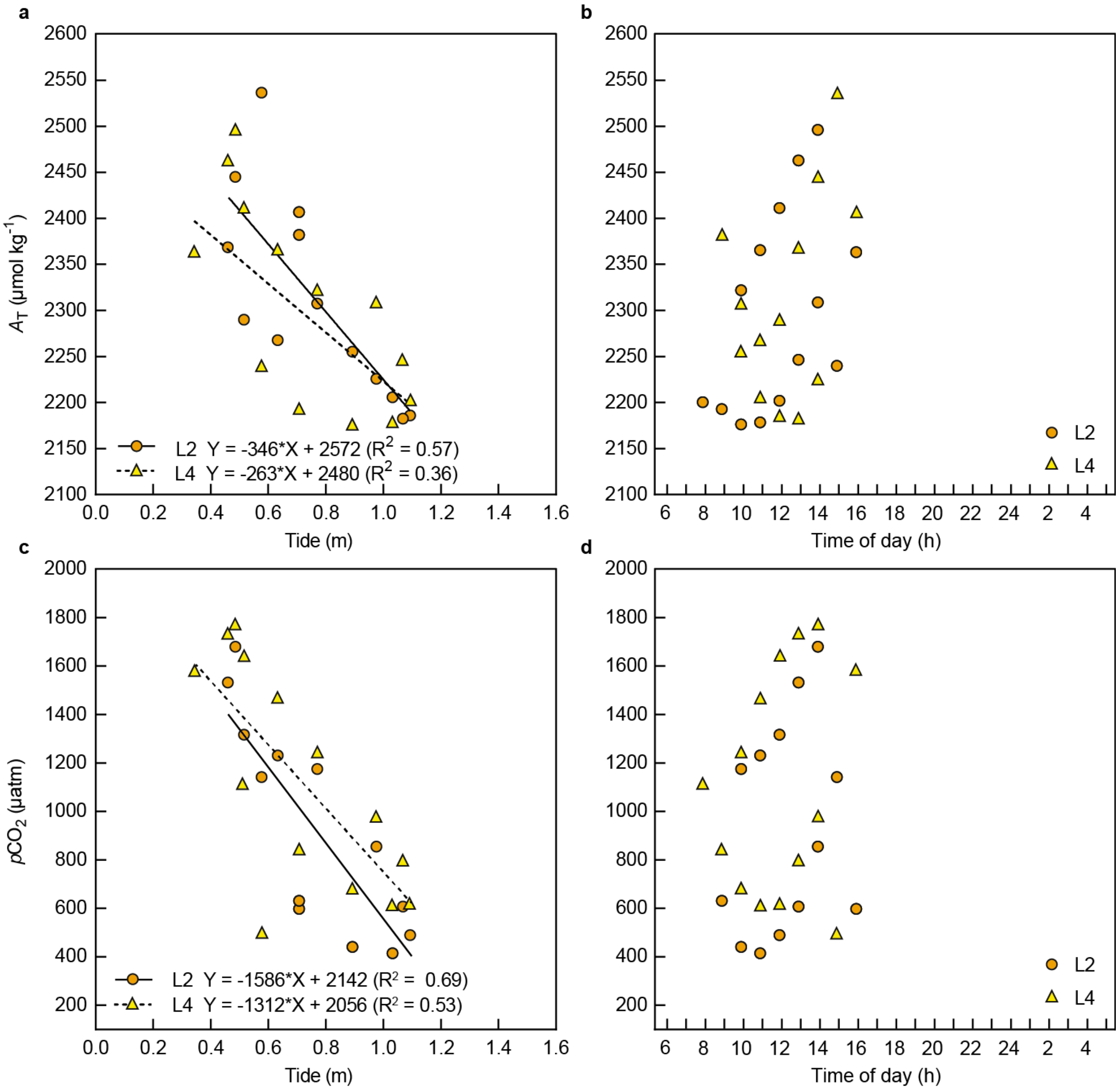

Figure S.5| Linear relationships between tidal height, alkalinity and *p*CO_2 for the Bouraké lagoon sites. Total alkalinity (A_T) against tide (**a**) and local time of day (**b**), and *p*CO_2 against tide (**c**) and local time of day (**d**) for lagoon sites L1 (circles) and L2 (triangles). Linear regressions significantly different from zero ($p < 0.05$) are indicated by solid lines (for L2) and dashed lines (for L4) with corresponding equations and R^2 values (see Supplementary Table 2 for full statistics).

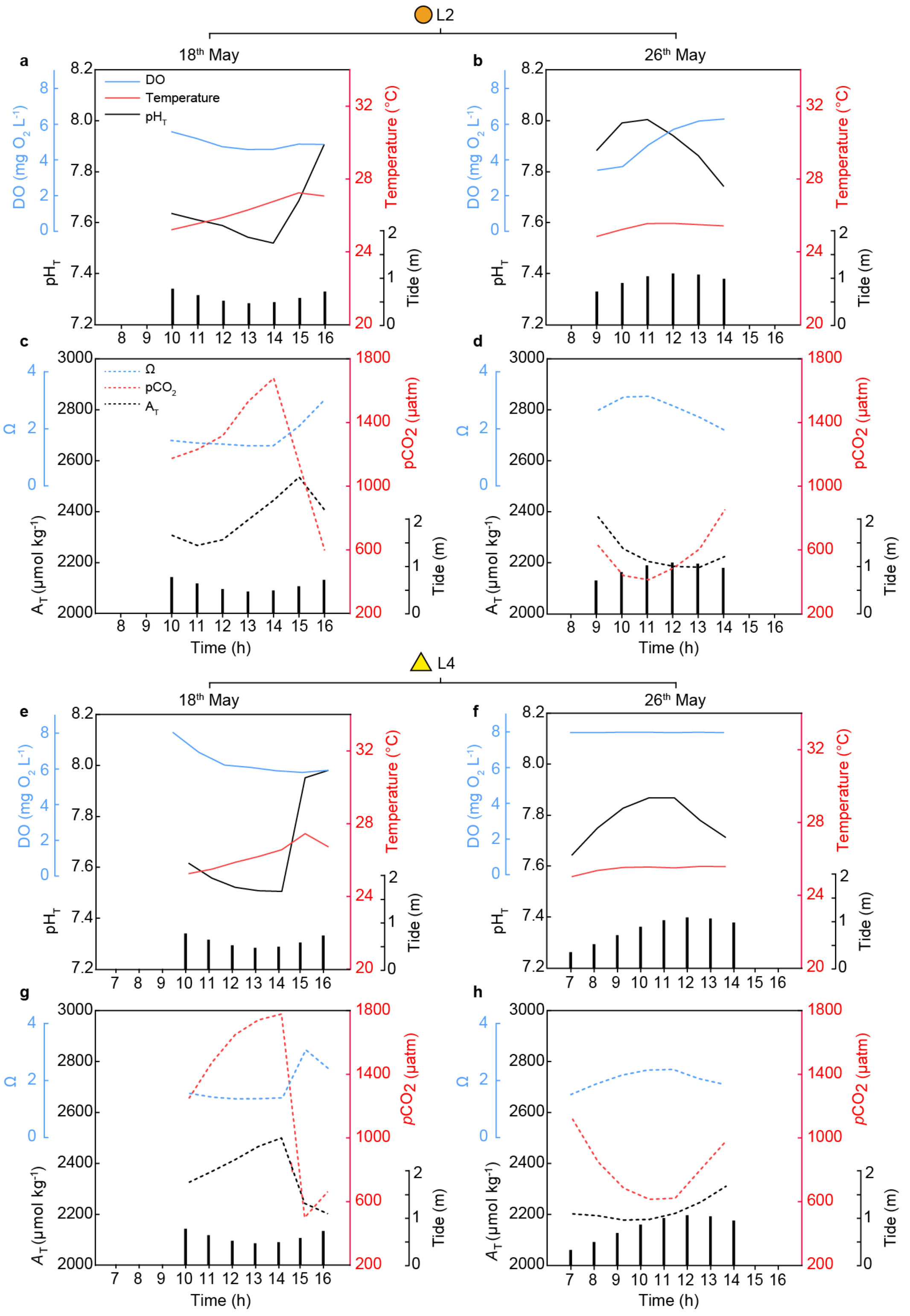

Figure S.6| Bouraké lagoon day-time carbonate chemistry. Two diel-cycles in May 2016 (third sampling period) were sampled to capture the possible diel and tidal cycles (see Methods). At the lagoon entrance (L4) and at one of the inner bay sites (L2) high-resolution (every-hour) total alkalinity (µmol kg^{-1}; A_T) samples were collected and coupled with *in situ* total pH (pH_T) (SeaFET^{TM}), salinity and temperature (°C) (YSI 660), and depth (*ca*. 1 m) as a proxy for pressure to calculate the daily carbonate chemistry of the lagoon system using CO2SYS[1] (L2 n = 13, L4 n = 14 sampling time points). Equilibrium chemical thermodynamic equations and the dissociation constants of ref. 2, ref. 3 for $KHSO_4$, and ref. 4 for boric acid.

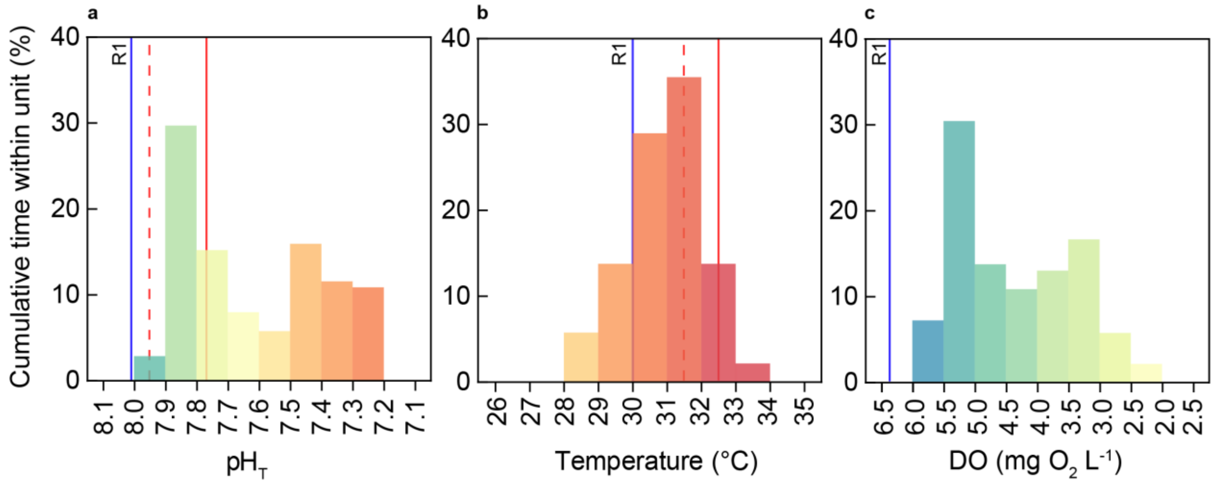

Figure S.7| Cumulative time of physico-chemical variables (pH_T, temperature and dissolved oxygen (DO) for the lagoon site L1. Data was collected from a 3-day sampling period in February 2016. Bars represent the cumulative time (y axes, %) where the physico-chemical conditions of the site are within the corresponding unit (x axes). Intergovernmental Panel on Climate Change (IPCC) estimates for pH_T and temperature at the end of the century under scenarios RCP 4.5 (dashed red lines) and RCP 8.5 (solid red lines) are indicated, as are the means for reference site 1 (R1, blue line).

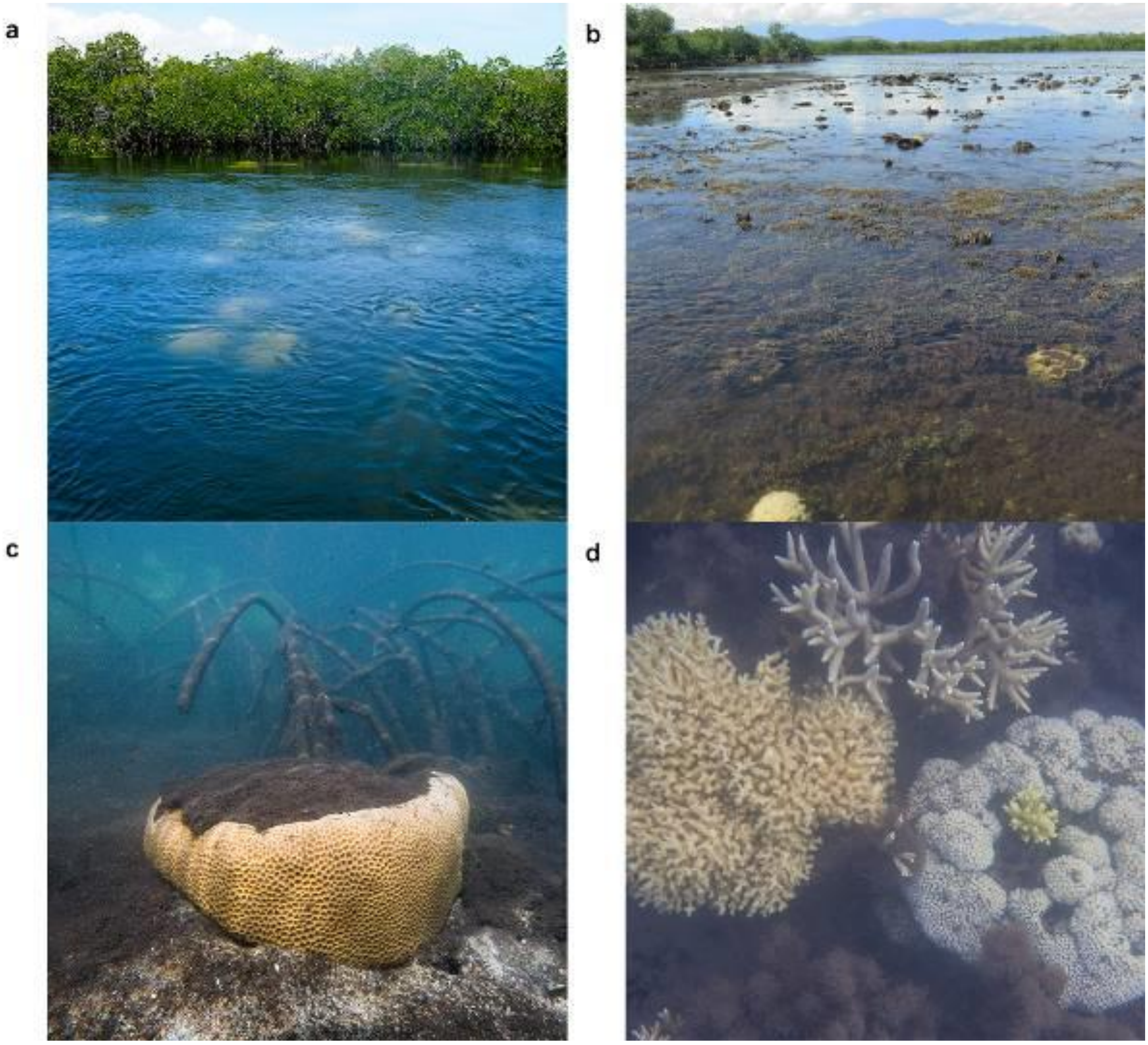

Figure S.8| Example of coral cover and diversity at the Bouraké lagoon. a, b, Coral colonies can be observed just under the surface, spanning the bay up to the mangrove trees. **c, d,** examples of the coral diversity and distribution within the lagoon.

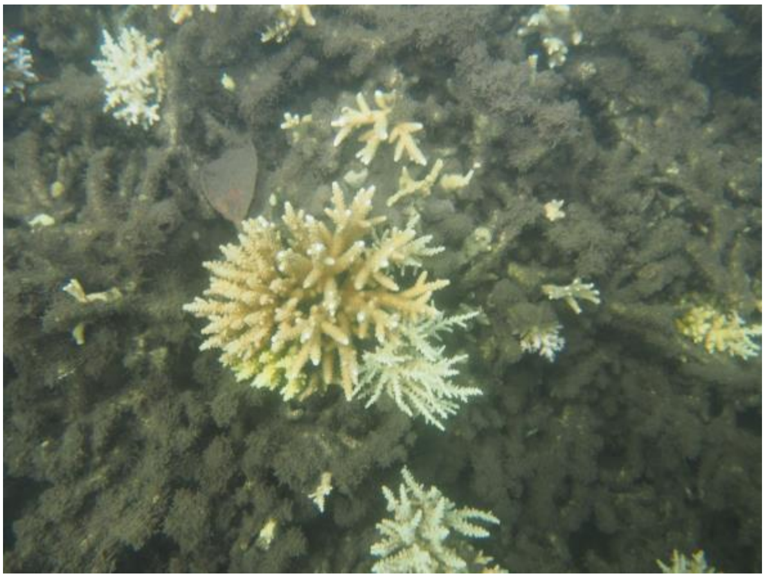

Figure S.9| Example of the old dead coral benthic substrate and new coral recruits in the Bouraké lagoon. The picture is from site L2 and demonstrates the framework available for coral recruitment. The dead coral structure is covered in fine sediment and organic detritus from the mangroves.

Supplementary Information: Tables

Table S.1| Statistical analysis of the physico-chemical parameters of the Bouraké lagoon sites (L1 and L2) relative to the reference sites (R1 and R2). Three-days of data were used from the second-sampling period to balance the three-days of data collected during the first-sampling period (matched lunar cycle between sampling periods, see Figure S.1). * $p < 0.05$, ** $p < 0.01$, *** $p < 0.001$.

**Sampling Period**	**Type**	***t*-value (df)**	***p*-value**
**pH_T**			
February	R1 vs L1	$t_{(142)} = 21.30$	$p < 0.001$***
March	R2 vs L2	$t_{(181)} = 43.44$	$p < 0.001$***
**Temperature**			
February	R1 vs L1	$t_{(172)} = 19.05$	$p < 0.001$***
March	R2 vs L2	$t_{(247)} = 6.42$	$p < 0.001$***
**Oxygen**			
February	R1 vs L1	$t_{(200)} = 23.47$	$p < 0.01$**
March	R2 vs L2	$t_{(248)} = 16.84$	$p < 0.001$***

Table S.2| Statistical results showing the effect of time-of-day and tidal-cycle on the physico-chemical parameters of the Bouraké lagoon sites (L1 and L2) and reference reef sites (R1 and R2). Linear models (y= bo+b_1x) fitted on the main physico-chemical parameter (pH_T, temperature, and dissolved oxygen) measured across the first (February, 2016) and second (March, 2016) sampling periods. Models were fitted for daytime and nighttime data. Goodness of fit was determined from statistical significance (* $p < 0.05$, ** $p < 0.01$, *** $p < 0.001$. and ns = non-significant) and R^2 (see Supplementary Figures 2-4 for R^2 and equations).

**Sampling Period**	**Site**	**Type**	**Parameters and Linear Regression test statistics**					
			**pH_T**		**Temperature**		**DO**	
			***F* (d.f.)**	***p*-value**	***F* (df)**	***p*-value**	***F* (df)**	***p*-value**
February	L1	Day	$F_{(1, 64)} = 0.55$	p = ns	$F_{(1, 64)} = 134.50$	$p < 0.001$***	$F_{(1, 64)} = 0.12$	p = ns
		Night	$F_{(1, 70)} = 0.13$	p = ns	$F_{(1, 70)} = 210.40$	$p < 0.001$***	$F_{(1, 70)} = 91.08$	$p < 0.001$***
		Tide	$F_{(1, 67)} = 126.80$	$p < 0.001$***	$F_{(1, 67)} = 0.48$	p = ns	$F_{(1, 67)} = 39.35$	$p < 0.001$***
	R1	Day	$F_{(1, 64)} = 5.11$	$p < 0.05$*	$F_{(1, 64)} = 63.73$	$p < 0.001$***	$F_{(1, 64)} = 23.76$	$p < 0.001$***
		Night	$F_{(1, 70)} = 2.70$	p = ns	$F_{(1, 70)} = 43.68$	$p < 0.001$***	$F_{(1, 70)} = 145.50$	$p < 0.001$***
		Tide	$F_{(1, 67)} = 0.26$	p = ns	$F_{(1, 67)} = 0.96$	p = ns	$F_{(1, 67)} = 0.03$	p = ns
March	L2	Day	$F_{(1, 64)} = 0.21$	p = ns	$F_{(1, 64)} = 224.60$	$p < 0.001$***	$F_{(1, 64)} = 2.59$	p = ns
		Night	$F_{(1, 70)} = 1.03$	p = ns	$F_{(1, 70)} = 192.61$	$p < 0.001$***	$F_{(1, 70)} = 1.98$	p = ns
		Tide	$F_{(1, 67)} = 28.58$	$p < 0.001$***	$F_{(1, 67)} = 3.80$	p = ns	$F_{(1, 67)} = 14.99$	$p < 0.001$***
	R2	Day	$F_{(1, 64)} = 26.74$	$p < 0.001$***	$F_{(1, 64)} = 528.62$	$p < 0.001$***	$F_{(1, 64)} = 129.41$	$p < 0.001$***
		Night	$F_{(1, 70)} = 23.75$	$p < 0.001$***	$F_{(1, 70)} = 141.50$	$p < 0.001$***	$F_{(1, 70)} = 335.31$	$p < 0.001$***
		Tide	$F_{(1, 67)} = 17.90$	$p < 0.001$***	$F_{(1, 67)} = 4.66$	$p < 0.05$*	$F_{(1, 67)} = 14.96$	$p < 0.001$***

Table S.3| Bouraké lagoon day-time carbonate chemistry variability collected over two diel-cycles in May 2016. At the lagoon entrance (L4) and at the two inner bays (L1, L2) hourly total alkalinity (A_T) samples were collected at *ca.* 1 m depth and coupled with high-resolution (30-min interval) *in situ* total pH (in total scale, pH_T), using SeaFET^{TM}, salinity and temperature, using YSI 660. The remaining seawater carbonate variables were calculated using CO2SYS[1] (see Methods). Data for A_T are n = 13, and 14 for L2 and L4 respectively; n = 197 for all the remaining variables.

Physico-chemical variable	Value	Site	
		**L2**	**L4**
pH_T	mean (± s.e.)	7.760 (0.01)	7.731 (0.01)
	max.	8.008	7.731
	min.	7.436	7.449
A_T (µmol kg^{-1})	mean (± s.e.)	2312.3 (30.20)	2297.6 (29.07)
	max.	2536.5	2296.3
	min.	2182.6	2176.4
Ω_{arg}	mean (± s.e.)	2.1 (0.05)	1.98 (0.04)
	max.	3.3	2.0
	min.	1.1	1.1
pCO_2 (µatm)	mean (± s.e.)	947 (31.4)	1010 (32.90)
	max.	2143	1010
	min.	415	435
HCO_3^- (µmol kg^{-1})	mean (± s.e.)	1980.4 (12.51)	1982.2 (12.51)
	max.	2361.1	1982.2
	min.	1700.7	1683.2
CO_3^{2-} (µmol kg^{-1})	mean (± s.e.)	135.7 (2.95)	126.03 (2.95)
	max.	209.2	126.0
	min.	68.6	71.9

Table S.4| Benthic data and statistical analysis for the Bouraké lagoon and reference reef sites. Mean (± s.e.) relative % cover of the benthic composition across the reference reefs (R1, R2) and Bouraké lagoon (L1-L4) sites (R1 $n = 4$; $n = 3$ for R2, and L1-L4). All data was collected over a three-day period during February 2016 (1st sampling period). Coral species observed while exploring sites that were not found on transects were also counted to provide a more complete overview of species diversity. Significant differences were assessed using Kruskal-Wallis test *post-hoc* Dunn's multiple comparisons test. Means with different letters (*a, b, c, d*) were significantly different. Benthic classification was conducted as per ref. 4. Non-scleractinian coral included soft-coral and anemones.

**Benthic component**	**Reference reef (%)**		**Bouraké lagoon (%)**				**Kruskal-Wallis H Test**	
	**R1**	**R2**	**L1**	**L2**	**L3**	**L4**	**H statistic**	***p*-value**
**Abiotic‡**	**85.9 (2.06) *a***	**5.9 (1.69) *b***	**74.6 (1.07) *ab***	**64.8 (0.43) *ab***	**55.9 (3.09) *ab***	**46.9 (2.69) *ab***	**H(6) = 15.63**	**$p < 0.05$**
Rock	19.4 (4.38)	9.5 (1.37)	2.3 (2.30)	0.6 (0.6)	21.3 (2.66)	18.9 (2.49)		
Rubble	62.7 (4.72)	17.0 (4.21)	0.1 (0.07)	(0.1) (0.1)	13.3 (3.15)	6.2 (3.08)		
Sand	3.7 (1.17)	0.9 (0.25)	0.0 (0.00)	0.0 (0.00)	6.6 (4.90)	13.1 (3.46)		
Dead coral	0.0 (0.00)	1.9 (0.67)	3.6 (2.43)	0.2 (0.17)	3.0 (1.76)	0.3 (0.30)		
Sediment	0.0 (0.00)	0.1 (0.13)	68.9 (21.9)	63.9 (4.52)	11.8 (3.01)	8.5 (4.13)		
**Algae**	**3.4 (0.40) *a***	**12.9 (0.50) *a***	**1.1 (0.20) *a***	**0.4 (0.14) *a***	**17.4 (4.26) *a***	**0.5 (0.25) *a***	**H(6) = 15.75**	**$p < 0.05$**
Non-calcifying filamentous-algae	3.4 (0.40)	12.3 (2.07)	1.0 (0.58)	0.4 (0.40)	10.6 (10.3)	0.5 (0.25)		
Calcareous algae	0.0 (0.00)	0.5 (0.48)	0.0 (0.00)	0.0 (0.00)	0.0 (0.00)	0.1 (0.03)		
Non-calcifying macro-algae	0.0 (0.00)	0.1 (0.07)	0.1 (0.07)	0.1 (0.03)	6.8 (2.48)	0.0 (0.00)		
**Scleractinian coral**	**10.3 (2.50) *a***	**55.0 (0.99) *b***	**24.3 (2.04) *ab***	**34.6 (3.93) *ab***	**26.7 (7.89) *ab***	**5.8 (0.79) *a***	**H(6) = 15.72**	**$p < 0.05$**
**Non- scleractinian coral**	**0.5 (0.29) *a***	**0.0 (0.00) *a***	**0.0 (0.00) *a***	**0.0 (0.00) *a***	**0.0 (0.00) *a***	**46.7 (11.09) *b***	**H(6) = 14.51**	**$p < 0.05$**
**Porifera cover**	**0.0 (0.00) *a***	**2.8 (1.40) *b***	**0.1 (0.03) *ab***	**0.2 (0.17) *ab***	**0.0 (0.00) *ab***	**0.1 (0.03) *ab***	**H(6) = 11.91**	**$p < 0.05$**
**Number of coral species** On transect (off transect)	39 (12)		14(7)					

‡Abiotic categories were modified to better distinguish the abiotic substrate of each site. The description of dead-coral refers to old mortality, as no transitional or recent mortality was observed. The categories were defined as follows: Rock, natural solid hard-ground that is not dead-coral; Rubble, fragmented/unconsolidated rock; Sand, Loose granular substrate (e.g. unconsolidated), typically pale brown; Dead-coral, any non-living parts of the coral in which the corallite structures are: i) covered over by organisms that are not easily removed; or ii) the overgrowing organisms (and perhaps the outer corallite structures) have been removed by a scraping herbivore or abraded by a storm, exposing the underlying skeleton; Sediment, finest abiotic substrate can appear consolidated into a congealed mass.

Table S.5| Scleractinian species list for the Bouraké lagoon and reference reef sites. Species identified on the 30 m continuous line transect and others observed while sampling but not found on the transects are indicated.

Bouraké lagoon		Reference reef sites	
**On transect**	**Off transect**	**On transect**	**Off transect**
Acropora aspera	*Acropora microphthalama*	*Acropora cf. acuminata*	*Acropora horrida*
Acropora sp.	*Acropora kirstyae*	*Acropora aspera*	*Acropora spp.*
Acropora formosa	*Cyphastrea sp.*	*Acropora sp.*	*Galaxea fascicularis*
Acropora pulchra	*Euphyllia cristata*	*Acropora florida*	*Pachyseris rugosa*
Acropora vaughani	*Fungia sp.*	*Acropora formosa*	*Pachyseris speciosa*
Acropora spp.	*Pavona decussata*	*Acropora gemmifera*	*Pavona cactus*
Coelastrea aspera	*Porites lobata*	*Acropora humilis*	*Pavona decussata*
Galaxea fascicularis		*Acropora kirstyae*	*Pectinia cf. alcicornis*
Goniastrea favulus		*Acropora latistella*	*Pectinia paeonia*
Montipora digitata		*Acropora cf. longicyathus*	*Porites lobata*
Pocillopora damicornis		*Acropora microphthalama*	*Psammocora contigua*
Porites cylindrica		*Acropora millepora*	*Turbinaria stellulata*
Porites lutea		*Acropora muricata*	
		Acropora nobilis	
		Acropora polystoma	
		Acropora pulchra	
		Acropora valida	
		Acropora vaughani	
		Acropora spp.	
		Coelastrea aspera	
		Favites spp.	
		Fungia spp.	
		Goniastrea favulus	
		Goniastrea pectinata	
		Isopora palifera	
		Lobophyllia corymbosa	
		Montipora aequituberculata	
		Montipora digitata	
		Montipora cf. nodosa	
		Montipora spp.	
		Pavona clavus	
		Pectinia lactuca	
		Pocillopora damicornis	
		Pocillopora verrucosa	
		Porites cylindrica	
		Porites lutea	
		Porites rus	
		Stylophora pistillata	

Table S.6| *In vitro* assessment of the coral metabolic parameters in the Bouraké lagoon and reference reef sites. Light and dark net calcification, net photosynthesis, and respiration rates measured on samples collected in the Bouraké lagoon (L1, L2) and reference reef (R1, R2) sites. Data are mean ± standard error (*n* = 4). Data was collected during the first sampling trip (February 2016).

Coral species	Light calcification (µmol $CaCO_3$ cm^{-2} h^{-1})				Dark calcification (µmol $CaCO_3$ cm^{-2} h^{-1})				Net photosynthesis (µmol O_2 cm^{-2} h^{-1})				Respiration (µmol O_2 cm^{-2} h^{-1})			
	L1	L2	R1	R2	L1	L2	R1	R2	L1	L2	R1	R2	L1	L2	R1	R2
Acropora pulchra	0.46 (0.08)		0.92 (0.09)		0.27 (0.03)		0.41 (0.05)		1.24 (0.16)		1.91 (0.14)		1.93 (0.13)		1.25 (0.11)	
Porites lutea	0.34 (0.17)		0.67 (0.11)		0.14 (0.04)		0.35 (0.07)		0.81 (0.18)		1.63 (0.24)		1.32 (0.24)		1.19 (0.16)	
Acropora formosa		0.81 (0.14)		1.06 (0.17)		0.30 (0.05)		0.39 (0.08)		0.95 (0.21)		1.46 (0.19)		1.98 (0.16)		1.14 (0.15)
Coelastrea aspera		0.39 (0.05)		0.62 (0.06)		0.21 (0.08)		0.33 (0.07)		0.92 (0.17)		1.07 (0.08)		1.05 (0.05)		0.66 (0.05)

Table S.7| Statistical analysis of the metabolic parameters of corals in the Bouraké lagoon (L1 and L2) compared to the reference reefs (R1 and R2). Each metabolic parameter: light calcification (G_L), dark calcification (G_D), photosynthesis (P) and respiration (R)**,** was compared between the lagoon and reference sites; *Acropora pulchra* and *Porites lutea*, sites R1 and L1; *A. formosa* and *Coelastrea aspera*, sites R2 and L2. * $p < 0.05$, ** $p < 0.01$, *** $p < 0.001$, ns = non-significant.

Species (Site)	Metabolism	*t*-value (d.f.)	*p*-value
Acropora pulchra (L1 *vs.* R1)	G_L	$t_{(6)} = 6.25$	ns
	G_D	$t_{(6)} = 2.33$	< 0.001***
	P	$t_{(6)} = 3.20$	< 0.05*
	R	$t_{(6)} = 3.95$	< 0.01**
Porites lutea (L1 *vs.* R1)	G_L	$t_{(6)} = 2.64$	< 0.05*
	G_D	$t_{(6)} = 4.73$	< 0.01**
	P	$t_{(6)} = 2.73$	< 0.05*
	R	$t_{(6)} = 0.46$	ns
Acropora formosa (L2 *vs.*R2)	G_L	$t_{(6)} = 1.28$	ns
	G_D	$t_{(6)} = 0.97$	ns
	P	$t_{(6)} = 1.79$	ns
	R	$t_{(6)} = 3.87$	< 0.01**
Coelastrea aspera (L2 *vs.* R2)	G_L	$t_{(6)} = 3.15$	< 0.05*
	G_D	$t_{(6)} = 0.20$	ns
	P	$t_{(6)} = 0.85$	ns
	R	$t_{(6)} = 5.27$	< 0.01**

Table S.8| Organic and Inorganic content of marine sediment for the Bouraké lagoon system (sites L1 and L2) and adjacent reference reef site (R2). Means ± standard error (*n* = 4) of organic carbon, sediment organic material (SOM) and sediment inorganic carbon (SIC). Organic carbon was determined using the modified Walkley-Black method as per ref. 6. SOM and SIC were determined by loss-on-ignition again following the method described by ref. 6.

Site	Organic Carbon (mg g^{-1})	SOM_{LOI} (g kg^{-1})	SIC_{LOI} (g kg^{-1})
L1	15.63 ± 2.02	62.08 ± 6.04	105.66 ± 2.41
L2	15.39 ± 2.20	62.01 ± 5.55	106.14 ± 1.70
R2	4.41 ± 0.21	30.49 ± 1.99	113.62 ± 1.52

Table S.9| Change in photosynthesis and calcification when abiotic substrate that was not live coral tissue was not covered by Parafilm. Means ± standard error (*n* = 10) of massive *Porites astreoides* incubated in the light. Coral were obtained from a shallow reef-crest and incubated as described in the methods section. Previously unpublished data (EF Camp) obtained from work in the Cayman Islands.

**Parameter**	**Abiotic substrate attached to coral colony**	
	**Covered**	**Not covered**
Calcification (μmol $CaCO_3$ cm^{-2} h^{-1})	0.81 (0.07)	0.75 (0.11)
Net Photosynthesis (μmol O_2 cm^{-2} h^{-1})	1.74 (0.09)	1.52 (0.14)

Supplementary Video Legends

Video 1: Underwater footage from the Bouraké semi-enclosed lagoon system at site 2 (L2). The footage was taken in February 2016.

Video 2: Underwater footage from Sainte-Marie Bay and the Bouraké semi-enclosed lagoon system at site 1 (L1) in March 2016. The footage shows the difference between sites of the extent of coral bleaching.

Supplementary References

1. Program developed for CO_2 system calculations (ORNL/CDIAC, 1998).
2. Lueker, T.J., Dickson, A.G., & Keeling, C.D. Ocean pCO_2 calculated from dissolved inorganic carbon, alkalinity, and equations for K1 and K2: validation based on laboratory measurements of CO_2 in gas and seawater at equilibrium. *Mar. Chem.* **70**, 105-119 (2000).
3. Dickson, A. G. Standard potential of the reaction: AgCl(s) + 1/2 H_2(g) = Ag(s) + HCl(aq), and the standard acidity constant of the ion HSO_4^- in synthetic seawater from 273.15 to 318.15 K. *J. Chem. Thermodyn.* **22**, 113–127 (1990).
4. Uppström, L.R. The boron/chlorinity ratio of deep-sea water from the Pacific Ocean. *Deep Sea Res.* **21,** 61–162 (1974).
5. Lang, C.J., *et al.* AGRRA Protocols Version 5.4, (2010); http://www.agrra.org/method/methodhome.html
6. Wang, X., Wang, J., & Zhang, J. Comparisons of three methods for organic and inorganic carbon in calcareous soils of Northwestern China. *PLoS ONE* **7**, e44334 (2012).
